# Effect of Long-Term Low-Dose Arsenic Exposure on DNA Methylation and Gene Expression in Human Liver Cells

**DOI:** 10.3390/ijms242015238

**Published:** 2023-10-16

**Authors:** Sandra Stößer, Tatjana Lumpp, Franziska Fischer, Sarah Gunesch, Paul Schumacher, Andrea Hartwig

**Affiliations:** Department of Food Chemistry and Toxicology, Institute of Applied Biosciences (IAB), Karlsruhe Institute of Technology (KIT), 76131 Karlsruhe, Germany

**Keywords:** arsenic, DNA methyltransferase, gene expression profiles, hypomethylation, cytotoxicity, cell cycle, HepG2

## Abstract

Millions of people around the world are exposed to elevated levels of arsenic through food or drinking water. Epidemiological studies have linked chronic arsenic exposure to an increased risk of several cancers, cardiovascular disease, central nervous system neuropathies, and genotoxic as well as immunotoxic effects. In addition to the induction of oxidative stress and inhibition of DNA repair processes, epigenetic effects, including altered DNA methylation patterns resulting in aberrant gene expression, may contribute to carcinogenicity. However, the underlying mechanisms by which chronic micromolar concentrations of arsenite affect the methylation status of DNA are not fully understood. In this study, human HepG2 hepatocarcinoma cells were treated with 0.5–10 μM sodium arsenite for 24 h, 10, or 20 days. During these periods, the effects on global DNA methylation, cell cycle phase distribution, and gene expression were investigated. While no impact on DNA methylation was seen after short-term exposure, global hypomethylation was observed at both long-term exposure periods, with concomitant induction of the DNA methyltransferase genes *DNMT1* and *DNMT3B*, while *DNMT3A* was slightly down-regulated. Pronounced time- and concentration-dependent effects were also seen in the case of genes involved in DNA damage response and repair, inflammation, oxidative stress response, and metal homeostasis. These results suggest that chronic low-dose arsenite exposure can lead to global hypomethylation. As an underlying mechanism, the consistent down-regulation of DNA methyltransferase genes could be excluded; alternatively, interactions at the protein level could play an important role.

## 1. Introduction

It is estimated that more than 100 million people worldwide are exposed to inorganic arsenic through drinking water or food, and there is high concern for adverse health effects [1,2]. On the other hand, arsenic trioxide (ATO) has been established as a therapeutic agent for acute promyelocytic leukemia (APL) [3]. From a toxicological point of view, epidemiological studies suggest that chronic low-dose exposure to inorganic arsenic in humans is significantly associated with an increased risk of lung, skin, and bladder cancer, central nervous system neuropathies, and immunotoxicity [1,2]. 

Arsenic and its inorganic compounds have been classified as carcinogenic to humans (Group 1) by both the German MAK Commission and the International Agency for Research on Cancer [4,5]. Although inorganic arsenic is a known carcinogen, it is not a strong mutagen. However, it can act as a co-mutagen and interfere with the cellular response to DNA-damaging exposures, such as UV radiation and benzo[*a*]pyrene [4,6,7,8]. Current evidence suggests that the induction of oxidative stress and interactions with DNA repair processes play crucial roles [1]. Furthermore, various studies have observed widespread disruption of transcriptional activity following arsenic exposure, indicating that the expression of genes, for example, those involved in oxidative stress and the DNA damage response, is affected by arsenic exposure [7,9,10]. Arsenic also appears to be involved in the disturbance of DNA methylation reactions, which could explain the extensive changes in gene expression [11]. 

The methylation of cytosine at so-called CpG islands is a fundamental determinant of chromatin structure and particularly important for regulating transcriptional activity. Thus, long-term transcriptional silencing generally correlates with the extent of CpG island methylation near gene promoters. The global disruption of DNA methylation is lethal for mammals, while gene-specific DNA hypermethylation has been associated with the loss of expression of tumor suppressor genes. On the other hand, genome-wide hypomethylation has been found to accompany oncogenic transformation, leading to genetic instability and spontaneous mutations. In humans, DNA methylation is catalyzed by three methyltransferases (DNMTs), namely, DNMT1, DNMT3A, and DNMT3B. DNMT1 appears to be responsible for maintaining methylation patterns during replication, while DNMT3A and DNMT3B play crucial roles in early embryogenesis and X-chromosome inactivation [12,13].

The methylation of cytosine to 5-methylcytosine occurs via the transfer of a methyl group from S-adenosylmethionine (SAM) to the C5 position of cytosine in CpG dinucleotides. However, SAM is also required during arsenite metabolism following exposure. This suggests that in the presence of excess arsenite, SAM, which is normally used for cellular methylation reactions, is used by arsenite methyltransferase (AS3MT) for arsenic methylation. This may lead to the depletion of SAM in the cell and the accumulation of S-adenosylhomocysteine (SAH), which, in turn, may suppress SAM-dependent methyltransferase. Since arsenic metabolism mediated by AS3MT primarily occurs in the liver and possibly in other AS3MT-expressing cell types, this mechanism of DNMT inhibition would be expected to be limited to these cell types [7,11]. Potential arsenite-induced changes in DNA methylation patterns have been found to result in the suppression of genes involved in cell cycle regulation, such as *p21*, subsequently leading to abnormal cell proliferation [14]. In addition to altered DNA methylation patterns, ROS-induced cell signaling also appears to impact cell cycle progression and cell proliferation [15].

Since most cell culture studies have been performed after short-term exposure of up to 24 h, and epigenetic changes would rather be expected to be evident at later time points, the present study aimed to compare short-term and long-term exposure to arsenite in human liver cells. Special focus was placed on gene expression profiles, cell cycle regulation, and the effect of arsenite on DNA methylation in epigenetic regulation. We found that arsenite clearly activates the transcriptional response to oxidative stress, cell cycle control, metal homeostasis, and DNA damage response in short- and long-term experiments, with pronounced time-dependent effects in some gene clusters. Furthermore, significant changes in the expression of genes coding for DNA methyltransferases were observed after chronic arsenite exposure, accompanied by global hypomethylation.

## 2. Results

To investigate the effect of arsenite after short- and long-term treatment, in the first step, the cytotoxicity was assessed. HepG2 cells were incubated with NaAsO_2_ in a concentration range of 0.5 µM to 50 µM for 24 h. The specific cytotoxicity profile was also used to determine the exposure range for the long-term experiments. To gain additional insights into the transcriptional toxicity profile of NaAsO_2_, HT RT-qPCR analysis was performed in HepG2 cells. To examine the impact of arsenic on epigenetic mechanisms, in addition to the investigation of genes relevant for epigenetic modifications, the global methylcytosine content was determined by HPLC. Furthermore, the effect of NaAsO_2_ on cell cycle phase distribution was examined using flow cytometry. For short-term incubations, HepG2 cells were treated with corresponding concentrations of NaAsO_2_ for 24 h. The long-term experiments were scheduled to span a period of 10 and 20 days.

### 2.1. Cytotoxicity

**Figure 1:**
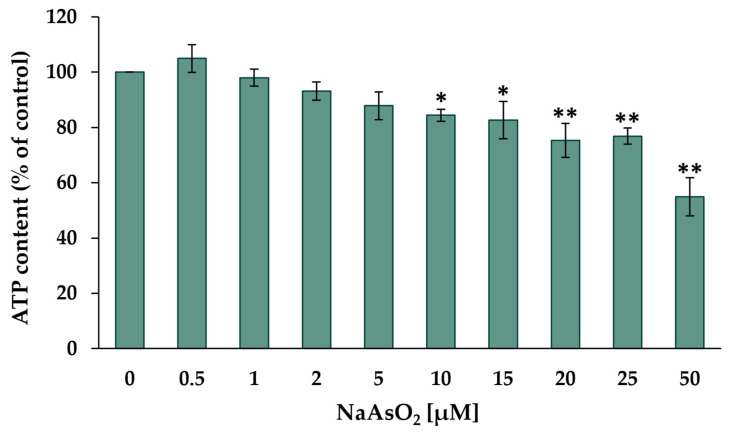
Cellular ATP content of HepG2 cells after 24 h of treatment with NaAsO_2_. Mean values ± standard deviations (SD) from three independent experiments are shown. Statistical analysis was performed to determine the differences between the exposed cells and the negative control using one-way ANOVA followed by Dunnett’s post hoc test: * (*p* ≤ 0.05), ** (*p* ≤ 0.01).

As the first step, the cytotoxic potential of arsenite was investigated, with the aim of establishing appropriate exposure concentrations for short- and long-term incubations within the non-cytotoxic concentration range. The cytotoxicity of NaAsO_2_ after 24 h of treatment was investigated in HepG2 cells using the CellTiter-Glo^®^ luminescence cell viability assay (Promega). In this test system, beetle luciferin is converted to oxyluciferin and photons. The luminescence produced correlates with the ATP content after complete lysis and thus reflects the number of viable cells. The results are shown in Figure 1. 

The incubation with low micromolar NaAsO_2_ concentrations did not show any effects on the cell viability of HepG2 cells. However, significant dose-dependent cytotoxic effects were observed starting at 10 µM NaAsO_2_, reducing the cellular ATP content by about 15%. Severe cytotoxicity was observed when HepG2 cells were treated with 50 µM NaAsO_2_, resulting in a 45% reduction in ATP content compared to the untreated control. Based on these data, a concentration range of 0.5 µM to 2 µM was selected for most long-term incubations, with an additional concentration range of up to 10 µM for the 20-day incubation period at some endpoints. The determination of cytotoxicity during the two long-term exposure periods was carried out via determination of the relative cell count (RCC) (Figure S1). A significant decrease in RCC was observed in both long-term exposure periods starting from a concentration of 5 µM NaAsO_2_. At the highest exposure concentration of 10 µM NaAsO_2_, this resulted in a 17% reduction in RCC after both 10-day and 20-day incubation periods. 

### 2.2. Gene Expression Analysis

**Figure 2:**
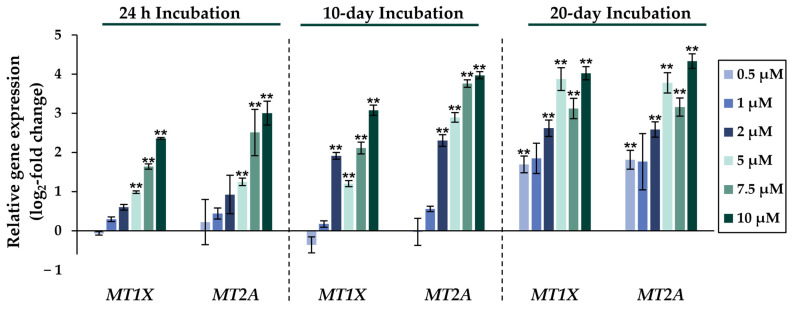
Impact of NaAsO_2_ exposure on the mRNA expression of metallothionein genes *MT1X* and *MT2A*, which are significantly altered in their expression in HepG2 cells. Cells were incubated with different concentrations of NaAsO_2_ for 24 h, 10 days, or 20 days. Results are presented as log_2_-fold changes in gene expression, compared to the respective untreated control. Shown are the means ± SD of three independent experiments each performed in duplicate. Statistical analysis was performed to determine the differences between the exposed cells and the negative control using one-way ANOVA followed by Dunnett’s post hoc test: ** (*p* ≤ 0.01).

**Figure 3:**
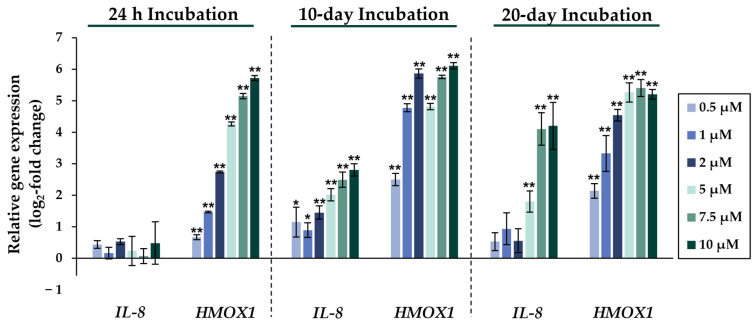
Impact of NaAsO_2_ exposure on the mRNA expression of *IL-8* and *HMOX1*, which are significantly altered in their expression in HepG2 cells. Cells were incubated with different concentrations of NaAsO_2_ for 24 h, 10 days, or 20 days. Results are presented as log_2_-fold changes in gene expression, compared to the respective untreated control. Shown are the means ± SD of three independent experiments performed in duplicate. Statistical analysis was performed to determine the differences between the exposed cells and the negative control using one-way ANOVA followed by Dunnett’s post hoc test: * (*p* ≤ 0.05), ** (*p* ≤ 0.01).

**Figure 4:**
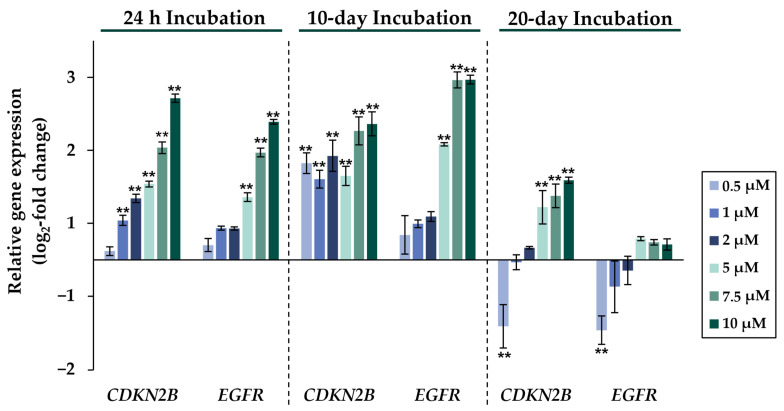
Impact of NaAsO_2_ exposure on the mRNA expression of the cell cycle-regulating genes *CDKN2B* and *EGFR*, which are significantly altered in their expression in HepG2 cells. Cells were incubated with different concentrations of NaAsO_2_ for 24 h, 10 days, or 20 days. Results are presented as log_2_-fold changes in gene expression, compared to the respective untreated control. Shown are the means ± SD of three independent experiments performed in duplicate. Statistical analysis was performed to determine the differences between the exposed cells and the negative control using one-way ANOVA followed by Dunnett’s post hoc test: ** (*p* ≤ 0.01).

**Figure 5:**
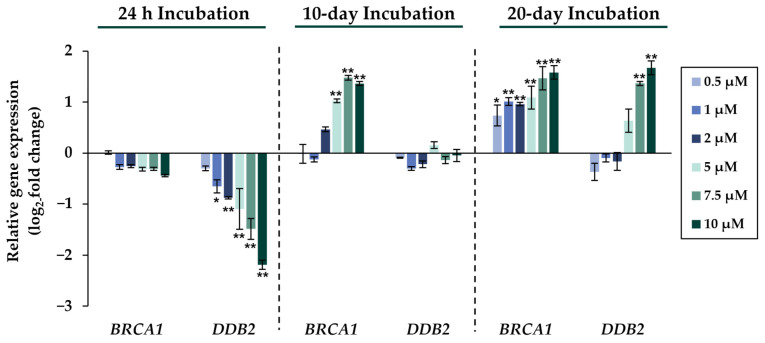
Impact of NaAsO_2_ exposure on the mRNA expression of DNA repair genes *BRCA1* and *DDB2*, which are significantly altered in their expression in HepG2 cells. Cells were incubated with different concentrations of NaAsO_2_ for 24 h, 10 days, or 20 days. Results are presented as log_2_-fold changes in gene expression, compared to the respective untreated control. Shown are the means ± SD of three independent experiments each performed in duplicate. Statistical analysis was performed to determine the differences between the exposed cells and the negative control using one-way ANOVA followed by Dunnett’s post hoc test: * (*p* ≤ 0.05), ** (*p* ≤ 0.01).

**Figure 6:**
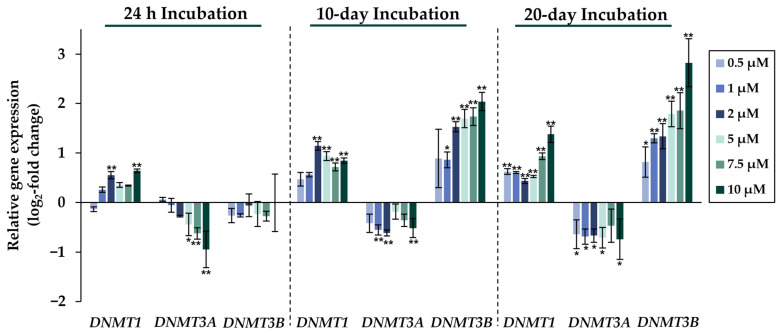
Impact of NaAsO_2_ exposure on the mRNA expression of genes involved in epigenetic regulation, *DNMT1*, *DNMT3A*, and *DNMT3B*, which are significantly altered in their expression in HepG2 cells. Cells were incubated with different concentrations of NaAsO_2_ for 24 h, 10 days, or 20 days. Results are presented as log_2_-fold changes in gene expression, compared to the respective untreated control. Shown are the means ± SD of three independent experiments each performed in duplicate. Statistical analysis was performed to determine the differences between the exposed cells and the negative control using one-way ANOVA followed by Dunnett’s post hoc test: * (*p* ≤ 0.05), ** (*p* ≤ 0.01).

To investigate the cellular response to NaAsO_2_ in HepG2 cells, gene expression profiles were established using high-throughput RT-qPCR. This method enabled the simultaneous assessment of the transcription of 95 genes in 96 samples. The gene set included markers associated with metal homeostasis, (oxidative) stress response, DNA damage and repair, cell cycle control, and apoptosis. A detailed description of this method has been previously published by our group [16]. Additionally, genes related to DNA methylation and histone modifications were included within the present study. Relative gene expression was calculated by normalizing the treated samples to the untreated control and expressed as log_2_ values. Reductions of at least 50% (log_2_-fold change ≤ −1) and duplications (log_2_-fold change ≥ 1) compared to the untreated control were considered relevant; in addition, concentration-dependent trends were also taken into account. The cells were incubated with NaAsO_2_ for 24 h, 10 days, or 20 days. A selection of significantly altered genes is depicted in Figure 2, Figure 3, Figure 4, Figure 5 and Figure 6. The log_2_ values for all genes are provided in Supplementary Table S1. The results for the entire gene panel, presented as heatmaps, are shown for the 24 h, 10-day, and 20-day incubations in Figures S2 and S3.

Comparing the gene expression profiles of HepG2 cells after 24 h, 10 days, and 20 days of exposure to NaAsO_2_, both dose- and time-dependent alterations in the transcriptional activity of a large number of genes were evident. As shown in Figure 2, the metallothionein-encoding genes *MTX1* and *MT2A* were significantly induced at all exposure times. The largest change, a 20-fold change, was observed for the *MT2A* gene after 20 days of incubation with 10 µM NaAsO_2_. Other examples of up-regulated genes include *HMOX1* and *IL-8*, which encode markers of inflammatory and oxidative stress responses (Figure 3). The most pronounced change was observed in the case of *HMOX1*, which already showed significant inductions in the sub-micromolar and low micromolar concentration range of arsenite throughout the incubation period. At the highest dose, the strongest concentration-dependent activation of *HMOX1* was observed after 10 days of incubation, with a 64-fold induction. Low micromolar concentrations of arsenite also resulted in significant inductions of the cell cycle- and apoptosis-regulating genes *EGFR* and *CDKN2B* following acute exposure. Exposure for 10 days further enhanced these effects, whereas after 20 days of exposure, inductions were only observed in the concentration range of 5 µM NaAsO_2_ and above. Sub-micromolar concentrations, on the other hand, led to the significant repression of both genes during the longest exposure time (Figure 4). Significant differences between the short-term and long-term incubations were observed for the DNA damage response and repair genes. As shown in Figure 5, after 24 h of incubation with NaAsO_2_, significant reductions of *DDB2* were already observed in the low micromolar concentration range, which increased with rising concentrations. At the highest dose, 4.2-fold suppression was observed. After longer incubation times, however, no impact was seen any more at 10 days incubation, and after 20 days incubation, even an induction was observed at 5 µM NaAsO_2_ and above. In addition to *DDB2*, *BRCA1* also showed a trend towards repression after 24 h incubation, but pronounced activation after long-term incubation of 10 or 20 days with NaAsO_2_, leading to significant inductions by sub-micromolar NaAsO_2_ concentrations after 20 days. Regarding genes involved in epigenetic alterations, significant concentration-dependent inductions of *DNMT1* and *DNMT3B* were detected at the two long-term exposure times (Figure 6). At the highest dose of 10 µM NaAsO_2_, 7-fold activation was detected after 20 days. On the contrary, *DNMT1A* showed significant repression after both the short-term incubation and the two long-term incubations.

### 2.3. Cell Cycle Regulation

**Figure 7:**
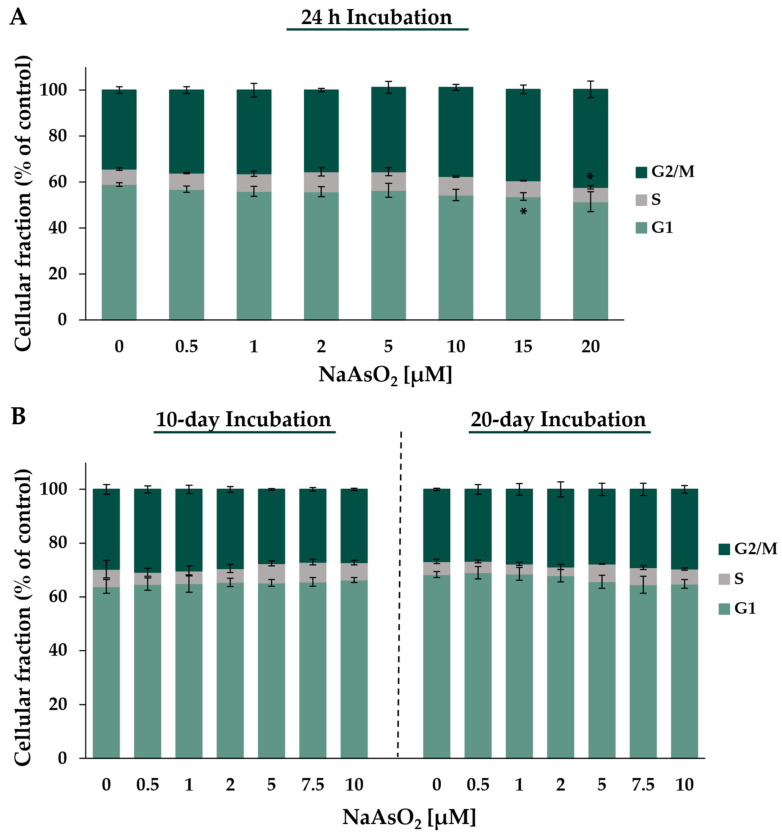
Analysis of cell cycle distribution of HepG2 cells after treatment with NaAsO_2_ for 24 h (**A**) and long-term incubation for 10 and 20 days (**B**). The cell cycle distribution was analyzed via DAPI staining using flow cytometry. Means ± standard deviations of three independent experiments are shown. Statistical analysis was performed to determine the differences between the exposed cells and the negative control using one-way ANOVA followed by Dunnett’s post hoc test: * (*p* ≤ 0.05).

Both short-term and long-term NaAsO_2_ treatments affected the expression of genes related to cell cycle regulation and proliferation. To assess the consequences at the functional level, the cell cycle distribution in HepG2 cells was analyzed via flow cytometry. Figure 7 shows the cell cycle phase distribution of HepG2 cells after 24 h (A) and after 10 or 20 days (B) of incubation with NaAsO_2_.

Considering the basal cell cycle distribution in control cells, the highest population was found in the G1 phase (~59%) and about 34% of the cells remained in the G2/M phase, while the S phase (~7%) represented the smallest cellular fraction. After 24 h of treatment with NaAsO_2_, a significant change in the cell cycle phase distribution compared to the basal distribution was observed, starting at a concentration of 15 µM. At the highest concentration of 20 µM NaAsO_2_, there was an 8.3% increase in cells in the G2/M phase with a simultaneous 7.5% decrease in cells in the G1 phase. Only the population in the S phase showed a similar level over the entire concentration range. After long-term incubations, treatment with NaAsO_2_ had no effect on the distribution of cell cycle phases throughout the applied concentration range. 

### 2.4. DNA Methylation

**Figure 8:**
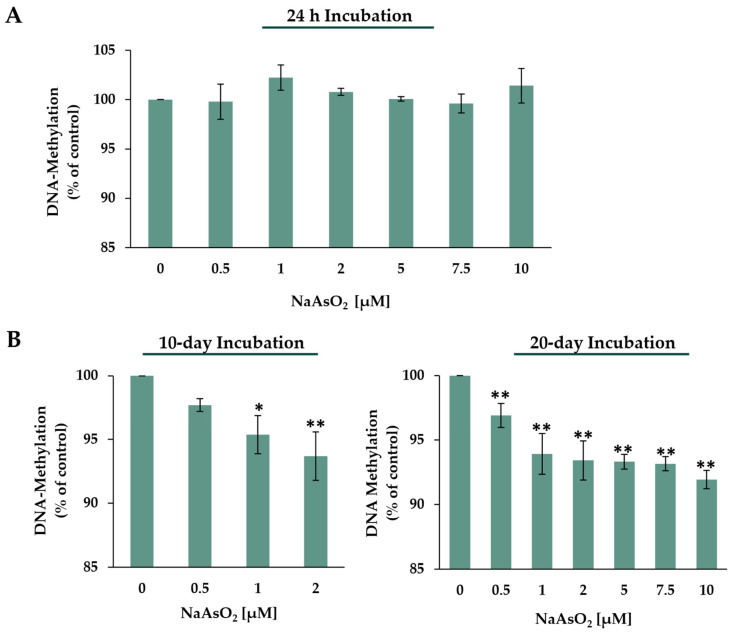
Effects of 24 h (**A**) and 10- or 20-day (**B**) NaAsO_2_ exposure on global DNA methylation in HepG2 cells, presented as global DNA methylation compared to the untreated control. Genomic 5-methyl-dCMP content was calculated as the proportion of total dCMP (5me-dCMP)/(5me-dCMP + dCMP) expressed in relation to the untreated control. Shown are the mean values ± SD of three independent experiments performed in duplicate. Statistical analysis was performed to determine the differences between the exposed cells and the negative control using one-way ANOVA followed by Dunnett’s post hoc test: * (*p* ≤ 0.05), ** (*p* ≤ 0.01).

Long-term incubation with NaAsO_2_ showed concentration-dependent induction of the genes *DNMT1* and *DNMT3B*, which are particularly involved in DNA methylation. To follow this impact at the functional level, global DNA methylation was investigated via HPLC-UV analysis. The amount of methyl-CMP present in each sample was expressed relative to the total dCMP present (methyl-dCMP + dCMP) and relative to the untreated control levels. Figure 8A shows the results after 24 h of short-term incubation and Figure 8B after the corresponding long-term incubations.

Short-term incubation with NaAsO_2_ did not significantly alter the global methylation level in HepG2 cells. However, regarding the two long-term incubation periods of 10 and 20 days, exposure to NaAsO_2_ caused significant and dose-dependent global hypomethylation. Already, in the low micromolar concentration range, starting at 1 µM, a significant decrease in 5-methyl-dCMP levels was observed after 10 days of exposure compared to the control. At the highest concentration of 2 µM, 10-day exposure to NaAsO_2_ resulted in a 6.3% decrease in 5-methyl-dCMP levels. In the 20-day long-term incubation, significant concentration-dependent decreases in global methylation were observed even at 0.5 µM. The highest incubation concentration of 10 µM NaAsO_2_ resulted in an 8% decrease in detectable 5-methyl-dCMP levels.

## 3. Discussion

For arsenic, it is well established that the induced carcinogenic effects occur upon sustained exposure over extended periods. As a result, long-term exposures in in vitro studies also appear to be of particular interest. Complementary to already existing long-term in vitro studies, it was the aim of the present work to investigate the toxicological and epigenetic effects of sub-micromolar to low micromolar, and thus, non-cytotoxic concentrations of arsenite following short- and long-term exposure in human liver cells. The applied concentrations are considered relevant for human exposure levels when compared to urine levels of inorganic arsenite. Even though the reference values, and thus, background levels of the general population are usually lower (for example, 15 µg/L urine (200 nM) in Germany), in occupational settings, levels up to 340 µg/L (4.5 µM) have been reported [17]. In Bangladesh, due to the highly elevated levels of inorganic arsenic in drinking water, even levels of up to 2166 µg/L urine (29 µM) were reported [18]. 

In the present study, it was observed that chronic exposure to sub-micromolar concentrations of arsenite resulted in global DNA hypomethylation and significant alterations in various gene expression clusters.

Altered epigenetic modifications, particularly DNA methylation, are believed to represent one underlying mechanism of arsenic-induced carcinogenesis. One potential cause of global hypomethylation could be the depletion of S-adenosylmethionine (SAM), although this relationship has not been fully elucidated. The enzymatic metabolism of inorganic arsenic in the liver to the methylated intermediates, monomethylarsonic acid (MMA(III)) and dimethylarsinic acid (DMA(III)), is mediated by S-adenosylmethionine-dependent arsenic methyltransferase (As3MT). However, SAM is also a co-factor that is generally required for cellular methylation reactions, including DNA methylation [11,19,20,21]. To further investigate the potential effect of arsenite metabolism on DNA methylation following long-term incubation, we employed the human liver cell line HepG2, which is proficient in As3MT [22,23,24].

The acute cytotoxic potential of NaAsO_2_ was assessed by determining the intracellular ATP levels after 24 h of exposure. At 10 µM NaAsO_2_, a significant decrease in basal ATP levels was evident, which further declined with increasing concentrations, reaching 45% after incubation with 50 µM arsenite. In the long-term experiments, a significant decrease in cell count was observed, starting at 5 µM NaAsO_2_ in both experimental periods. The cytotoxic effect of arsenite has been previously demonstrated in various cell lines, although the precise mechanisms remain incompletely understood [25,26]. One significant specific mechanism appears to be arsenite-induced cellular oxidative stress, leading to the inhibition of oxidative phosphorylation and a decline in cellular ATP levels [27].

To assess the effects of arsenite at the transcriptional level and to identify potential differences in gene expression profiles between short- and long-term exposed cells, gene expression analyses were performed using a high-throughput RT-qPCR method [16]. The most pronounced changes were observed in the cluster associated with the oxidative stress response in both short- and long-term arsenite treatments, particularly involving alterations in target genes of the KEAP1-NRF2 signaling pathway. The strongest induction was observed in the *HMOX1* gene, encoding heme oxygenase 1, which serves as a sensitive indicator for oxidative stress. Remarkably, a significant effect was already evident after 24 h even at the lowest sub-micromolar arsenite concentration, which was sustained in the long-term experiment. Other sensor genes for oxidative stress, such as *HSPA1A*, were strongly induced after short-term exposure, although to a lesser extent. However, this effect was not maintained in the long-term exposure, suggesting an early stress-induced response. Additionally, there were minor but dose-dependent inductions of *GCLC* and *TXNRD1*, which were significantly amplified after 10 days of exposure. Conversely, a declining trend of induction was observed at the longest incubation time of 20 days, indicating a time-dependent arsenite-induced response. *GCLC* and *TXNRD1* are also target genes of the NRF2 transcription factor, which regulates cellular levels of glutathione (GSH) and thioredoxin (TXN) as part of the cellular antioxidant response. The genes *NFKB1* and *NFKBIA*, whose expression is mediated by the redox-sensitive transcription factor NF-κB, exhibited mild repression throughout the entire exposure period. The arsenite-induced induction of genes involved in the oxidative stress response, as previously demonstrated in short-term experiments in other cell lines [28], appears to persist even in the long-term studies.

Remarkably, a significant concentration-dependent induction of interleukin-8 *(IL-8*) was observed only during the two long-term exposure periods, and not after short-term exposure. *IL-8* is a marker of inflammatory response, but plays also a crucial role in cell transformation, tumor growth, and metastasis [29,30]. For example, long-term exposure to arsenite has been shown to be involved in the transformation of human bronchial epithelial (HSE) cells, involving the HIF-2α-mediated up-regulation of *IL-6* and *IL-8* [30]. Therefore, IL-8 appears to play an important role in later steps of arsenite-induced carcinogenicity.

In addition to the induction of genes involved in the oxidative and inflammatory stress response, significantly increased expression levels of *MT1X* and *MT2A* were observed upon NaAsO_2_ exposure. These genes encode metallothioneins, indicative of elevated intracellular metal ion concentrations; their expression was significantly induced even at sub-micromolar concentrations in the long-term experiment. These results suggest the involvement of the zinc-binding, metal-regulatory transcription factor MTF-1, which is activated by the release of zinc from metallothionein (MT) upon metal overload or oxidative stress [31]. Interestingly, the up-regulation of *SLC30A1*, which encodes a proton-coupled zinc antiporter involved in the efflux of zinc from cells to prevent toxicity, was also observed with increasing exposure duration [32].

Concentration-dependent changes in gene expression profiles were also obtained for cell cycle- and apoptosis-related genes in the short-term incubations. Significantly increased inductions were observed for *CDKN2B*, which encodes the cell cycle regulator p15, as well as *EGFR* and *CDKN1A*. These changes in the gene expression profiles of cell cycle-regulating genes were maintained in the 10-day long-term exposure; interestingly, at this time point, the up-regulation of *CDKN2B* occurred at lower concentrations, while *EGFR* resulted in similar up-regulation as compared to 24 h incubation. The picture changed considerably after 20-day incubation: here, both *CDKN2B* and *EGFR* were down-regulated at low concentrations of arsenite, but up-regulated at higher concentrations, even though less pronounced when compared to the shorter incubation times. Time-dependent differences in EGFR-related effects were also reported in the literature. Thus, in human BEAS-2B cells, increased EGFR protein levels were observed after acute (24 h) and chronic (24 week) arsenite exposure, albeit involving different mechanisms. While the elevated expression of *TGFα* was observed after long-term treatment, accompanied by increased EGFR phosphorylation and elevated cell surface EGFR levels, no concomitant increase in *TGFα* expression was evident after 24 h, suggesting time-dependent differences in the molecular mechanisms of EGFR activation [33]. 

To investigate these different effects of arsenite on the mRNA expression of cell cycle-regulating genes at a functional level, flow cytometric analyses were conducted, also focusing on a comparison between short-term and long-term exposed cells. Acute treatment with NaAsO_2_ at a concentration of 15 µM or higher led to G_2_/M arrest, indicative of DNA damage after short-term exposure, in agreement with the results on the transcriptional level. This supports previous studies where pronounced G2/M arrests after acute arsenite exposure have been observed in various cell lines [15,34,35]. However, in the long-term experiments with 10- and 20-day sub-micromolar and micromolar NaAsO_2_ incubations, no effect on cell cycle progression was observed within the present study. In contrast, in a study by Ganapathy et al., a delayed exit from mitosis was demonstrated in BEAS-2B cells and keratinocytes following one month of chronic exposure to 0.5 µM sodium arsenite [36]. Time- and cell line-dependent effects may play a role in this discrepancy. Additionally, it should be noted that the flow cytometric analyses conducted in this study did not distinguish between G2 and M arrests.

Considerable differences in gene expression profiles between short- and long-term exposed cells were observed in genes involved in the DNA damage response and repair. As previously reported [37,38] and also seen within the present study, short-term incubation with arsenite resulted in the concentration-dependent repression of DNA repair factors involved in basically all major DNA repair pathways. However, opposite effects were observed in the long-term experiments, where significant inductions were observed, including the genes *BRCA1*, *DDB2*, and *LIG1* involved in double-strand break and nucleotide excision repair. These results suggest that the down-regulation of DNA repair may only be a transient reaction of the cell, compensated by the up-regulation of DNA repair genes upon chronic arsenite exposure. 

Significant differences were also observed in the cluster of genes associated with epigenetics between cells exposed to short- and long-term arsenite treatment. Since arsenite may interfere with DNA methylation, special focus was placed on the genes encoding DNA methyltransferases. Interestingly, in addition to time-dependent effects, pronounced differences were also seen between the expression patterns of the different DNA methyltransferases. In the case of *DNMT3A*, significant down-regulation was observed at all time points; this effect was observed even at very low concentrations after long-term exposure. In contrast, *DNMT1* showed moderate induction at all time points. The expression levels of *DNMT3B,* however, were not altered after 24 h of incubation, but were dose-dependently elevated after both 10 and 20 days of incubation, starting at sub-micromolar NaAsO_2_ concentrations. This supports our observation that changes in epigenetic modifications require long-term exposure conditions. The genes *DNMT1*, *DNMT3A*, and *DNMT3B* encode DNA methyltransferases, which perform different functions in the methylation process. The de novo DNA methylation during embryogenesis has been proposed to be carried out by the DNA methyltransferases DNMT3A and DNMT3B [39,40], while DNMT1 was thought to maintain specific methylation patterns in a replication-dependent manner [41,42]. However, an increasing number of studies suggest that the classification into de novo and maintenance DNA methyltransferases may be an oversimplification. Thus, DNMT3A and DNMT3B appear to also play important roles in the maintenance of methylation patterns [43,44], and DNMT1 is involved in establishing DNA methylation patterns, as well [45,46].

The pronounced induction of two DNMT-encoding genes, namely, *DNMT1* and *DNMT3B*, at the transcriptional level may suggest DNA hypermethylation. Therefore, the extent of global DNA methylation was determined via HPLC-UV. Acute arsenite exposure did not show any impact on global 5-mC content. Interestingly, however, 10-day long-term incubation with 1 µM already led to significant hypomethylation, which was further amplified with increasing concentration. This hypomethylation was also observed after 20-day exposure, even at the sub-micromolar concentration of 0.5 µM, with pronounced dose-dependency at higher arsenite concentrations. Altogether, long-term exposure to low concentrations of NaAsO_2_ resulted in global hypomethylation in human liver cells, which was not detected in short-term incubations.

Global hypomethylation can contribute to malignant transformation by promoting chromosomal defects that can lead to genetic instability and by promoting spontaneous mutations [11]. The induction of global hypomethylation due to long-term arsenite exposure confirms several observations reported in the literature [47,48,49]. However, controversial results were obtained with respect to the role of DNMT expression levels as one potential underlying mechanism of hypomethylation. Within the present study, down-regulation was only observed in the case of *DNMT3A*. In contrast, Rea et al. observed a notable reduction in the transcript levels of all *DNMTs* in BEAS-2B cells following 36-day exposure to 0.5 µM sodium arsenite, which also resulted in decreased protein levels of DNMT1, DNMT3A, and DNMT3B. Nevertheless, Zhao et al. demonstrated increased *DNMT* expression levels, Benbrahim-Tallaa et al. reported unchanged *DNMT* expression levels, and Cui et al. observed a reduction in *DNMT1* mRNA expression after 48 h of exposure of HepG2 cells to arsenite [19,47,48,49]. Therefore, data concerning the role of gene expression changes in global hypomethylation remain inconsistent at present. One other potential consequence of arsenite exposure consists in the consumption of S-adenosylmethionine (SAM) during arsenic metabolism, which, in turn, could lead to the accumulation of S-adenosylhomocysteine (SAH). Both the depletion of SAM and the accumulation of SAH can inhibit DNMT activity at both the mRNA and protein levels through negative feedback. It is important to note, however, that the inhibition of DNMT activity primarily occurs in the liver, and possibly in other cell types expressing As3MT, due to arsenic metabolism mediated by AS3MT. Therefore, this mechanism does not appear to be the general reason for arsenite-induced hypomethylation. In cells unable to metabolize arsenic, activation of the trans-sulfuration pathway as a consequence of oxidative stress appears to be a possible cause of global hypomethylation [11]. 

In addition to the indirect inhibition of methyltransferases through the limitation of the co-factor SAM, there is a possibility that direct inhibition at the protein level could be a cause of global hypomethylation. Potentially very sensitive targets are zinc-binding structures, so-called zinc fingers, where zinc complexes four amino acids, including two to four cysteines and up to two histidines. Interactions with these structures have been shown to be particularly relevant in arsenite-induced DNA repair inhibition [50]. Thus, the most sensitive target related to DNA repair affected by trivalent arsenicals is poly(ADP-ribosyl)ation, inhibited by low nanomolar concentrations of arsenite, MMA(III), and DMA(III). Furthermore, all three trivalent arsenicals inhibited isolated PARP1, indicating a direct interaction with this enzyme [25,51,52]. Also, trivalent but not pentavalent arsenicals have been shown to release zinc from a 37-amino-acid peptide resembling the zinc finger domain of the human XPA protein (XPAzf) [53], albeit via different mechanisms. Whereas equimolar concentrations of MMA(III) mediated zinc release, forming mono- and diarsenical derivatives of XPAzf, a 10-fold excess of arsenite was required to partially oxidize XPAzf, yielding one or two disulfide bonds [54]. Interestingly, zinc finger structures of the CXXC and ADD classes have also been identified in DNA methyltransferases [55,56]. The CXXC zinc finger structures are present in various DNA methyltransferases, including DNMT1, and play a crucial role in the recognition and binding of specific DNA sequences. They consist of conserved cysteine residues that coordinate zinc ions, forming a stable protein structure. It has been shown for DNMT1 that the binding of the CXXC zinc finger domain to CpG-rich DNA regions is essential for its catalytic activity [55,57]. The impact of arsenite on other DNA methyltransferases such as DNMT3A has also been investigated. DNMT3A contains an ADD domain with a zinc finger motif in its DNA binding domain, facilitating the establishment of new DNA methylation patterns during development and DNA repair. It has been demonstrated that arsenite directly binds to the cysteine-rich zinc-binding ADD domain of DNMT3A [58]. Finally, due to the complexation of zinc to cysteines within zinc-binding structures, they may also be particularly sensitive to oxidative stress induced by arsenite [55].

In summary, the existing evidence points to different cell-specific mechanisms of action of arsenic in DNMT activity and global DNA methylation patterns. According to the results obtained within the present study, hypomethylation appears not to be due to the inhibition of DNMT mRNA expression. Therefore, the ability of arsenite to influence methyltransferase activity in human liver cells may occur through the depletion of cofactors or direct inactivation of the respective proteins, potentially via interaction with zinc-binding structures present in DNMTs. This aspect needs to be further investigated. 

## 4. Materials and Methods

### 4.1. Materials

All chemicals were purchased from Sigma-Aldrich (Taufkirchen, Germany) or Carl Roth (Karlsruhe, Germany), the cell culture medium and additives were obtained from Sarstedt (Nuembrecht, Germany), and fetal bovine serum (FBS) was from Thermo Fisher Scientific (Dreieich, Germany). PCR consumables were purchased from Brand (Wertheim, Germany), and all cell culture materials were bought from Sarstedt (Nuembrecht, Germany). FACSFlow was acquired from BD (Heidelberg, Germany), and DAPI (CyStain^®^ DNA/Protein) was obtained from Sigma-Aldrich (Steinheim, Germany). PCR reagents were from Macherey-Nagel (Dueren, Germany), Fluidigm (San Francisco, CA, USA), Applied Biosystems (Forster City, WI, USA), Bio-Rad (Munich, Germany), New England Biolabs (Frankfurt am Main, Germany), and Teknova (Hollister, CA, USA). Primers for RTqPCR were synthesized by Eurofins (Ebersberg, Germany).

### 4.2. Cell Culture and Drug Treatment

The human liver cancer cell line HepG2 was cultured as a monolayer in Dulbecco’s modified Eagle’s medium (DMEM) supplemented with 10% heat-inactivated fetal bovine serum (FBS), 100 U/mL penicillin, and 100 µg/mL streptomycin. The cells were cultured at 37 °C with 5% CO_2_ in the air and 100% humidity. Logarithmically growing cells were incubated with NaAsO_2_ in the short-term experiments for 24 h, and in the long-term experiments for 10 or 20 days. For the long-term experiments of 10 or 20 days, the cells were passaged every 5 days and re-incubated with the arsenite-containing medium. The non-exposed control cells were concurrently cultivated and sub-cultured over the same period to discern the effects of continuous passaging from those of sustained exposure to arsenic.

### 4.3. Cytotoxicity Assays

For the analysis of ATP content, the CellTiter-Glo^®^ Luminescent Cell Viability Assay Kit (Promega GmbH, Walldorf, Germany) was used. Cell cultivation and incubation were performed in 96-well plates. Specifically, 3 × 10^4^ cells per well were seeded. Logarithmically growing cells were treated with NaAsO_2_ according to the instructions provided in the section on drug treatment. After removing the incubation medium, the cells were washed twice with PBS, and 100 µL of fresh medium was added to the wells. The CellTiter-Glo^®^ Luminescent Cell Viability Assay was performed according to the manufacturer’s instructions. The plate was equilibrated in the dark at room temperature for 30 min, and 100 µL of the CellTiter-Glo^®^ reagent was added. Chemiluminescence was measured after brief shaking and an additional 10 min stabilization period of the signal using the Infinite^®^ 200 Pro microplate reader (Tecan Group Ltd., Männedorf, Switzerland). The ATP content and, consequently, the decrease in cell viability were expressed as percentages of the untreated control cells.

### 4.4. High-Throughput RT-qPCR

For the gene expression analysis, 5 × 10^5^ cells were seeded. Logarithmically growing cells were treated with NaAsO_2_ for 24 h, 10 days, or 20 days. Subsequently, they were washed with PBS, trypsinized, resuspended in ice-cold PBS containing 10% FBS, and collected via centrifugation. RNA was then isolated using the MN NucleoSpin^®^ RNA Plus Kit (Macherey-Nagel, Düren, Germany) according to the manufacturer’s instructions. The subsequent high-throughput RT-qPCR was performed via Fluidigm Dynamic Arrays using the BioMark™ system, as previously described [16]. The original gene set was partly replaced with 16 genes of epigenetic regulation (*DNMT1*, *DNMT3A*, *DNMT3B*, *HDAC1*, *HDAC2*, *HDAC3*, *HDAC10*, *KDM3A, MeCP2*, *MBD4*, *SETD2*, *TET1*, *TET2*, *TET3*, *EHMT2*, *EP300*) and 4 genes of cell cycle control and senescence (*CDKN2A*, *CDKN2D*, *FOXO1*, *FOXO3*). All genes are presented in Table S1 and all data of the newly established primers are listed in Table S2. The data were analyzed using Fluidigm Real-Time PCR Analysis and GenEx software, version 5.3.6.170. Normalization was performed using up to five reference genes (*ACTB*, *B2M*, *GAPDH*, *GUSB*, and *HPRT1*). Changes in the transcriptional values of the target genes were presented as log_2_-fold change compared to the respective untreated controls, with the relative quantities calculated using the ΔΔCq method [59].

### 4.5. Analysis of Cell Cycle Distribution via Flow Cytometry

For the cell cycle analysis, 5 × 10^5^ cells were seeded. Logarithmically growing cells were treated with NaAsO_2_, as indicated in the section on drug treatment. Subsequently, both the culture medium and trypsinized cells were transferred to a 15 mL tube. After centrifugation, the supernatant was discarded, and the cells were resuspended in PBS.

For the subsequent cell cycle analysis, the cells were fixed with ice-cold 96% ethanol and stored at 20 °C overnight. The fixed cells were centrifuged, the supernatant was discarded, and the cells were washed with PBS and centrifuged again. The cells were then resuspended in a DAPI staining solution (Partec, Münster, Germany) and incubated on ice in the dark for 30 min. Fluorescence was measured using a BD LSRFortessa flow cytometer (BD, Heidelberg, Germany) with a violet laser at an excitation wavelength of 405 nm and a bandpass filter of 450/50 nm. To determine the cell cycle distribution, the fluorescence signal was plotted against cell count in a histogram.

### 4.6. Quantification of Methyl-dCMP Content

To quantify the global DNA methylation level, the total 5-methyl-cytosine content of the samples was analyzed. For this purpose, 5 × 10^5^ HepG2 cells were seeded for each sample. Logarithmically growing cells were treated with NaAsO_2_ as described in the section on drug treatment. Subsequent DNA isolation was performed using the Monarch^®^ Genomic DNA Purification Kit from New England BioLabs (Frankfurt am Main, Germany) following the manufacturer’s instructions. To analyze the individual nucleosides via HPLC, the isolated DNA had to be degraded. First, the concentration and purity of the DNA were determined. For this purpose, 2 μL of the sample was pipetted in duplicate into a microplate and spectrophotometrically measured at a ratio of 260/280 nm using the Tecan Infinite m200 PRO. A ratio between 1.8 and 2 indicated sufficient purity. Subsequently, the required volume of the eluate to obtain 1 μg of DNA was calculated. The Nucleoside Digestion Mix (New England Biolabs, Frankfurt am Main, Germany) was used for DNA degradation according to the manufacturer’s instructions. The HPLC analysis was performed using the Ultimate 3000 system from Thermo Scientific (Dreieich, Germany), an ultra-high-performance liquid chromatography (UHPLC) system. A Luna^®^ 5 μm C18(2) 100 Å, Liquid Chromatography Column 250 mm × 2 mm from Phenomenex was used, which is a reversed-phase column with a stationary phase of silica gel, modified with octadecylsilane and trimethylchlorosilane endcapping. The mobile phase consisted of 77% distilled water, 15% 50 mM sodium acetate trihydrate buffer at pH 4, and 8% methanol as an organic modifier, and the separation was performed isocratically. A UV detector was applied, with the cytidine nucleoside measured at 270 nm and 5-methylcytidine at 280 nm. Prior to sample measurement, 7-point calibration was performed using cytidine in a concentration range of 4–16 μM and 5-methylcytidine in a concentration range of 0.1–0.7 μM. The content of methyl-CMP in each sample was calculated according to the following formula and compared to the untreated control:

$$\mathrm{Genomic}\;\text{5-methyl-cytosine}\;\left(\%\right)=\frac{n\;\left(\text{5-methyl-cytidines}\right)}{n\;\left(\text{5-methyl-cytidines}\right)+n\;\left(\mathrm{cytidines}\right)}$$

### 4.7. Statistical Analysis

All cell culture experiments were conducted in duplicate in at least three independent experiments, using three different cell passages. Gene expression experiments were performed in at least three individual experiments, with two technical replicates each. The data are presented as mean ± standard deviation. Differences between untreated and arsenite-treated samples were evaluated using one-way ANOVA followed by Dunnett’s post hoc test. *p*-values below 0.05 (0.01) were considered statistically significant. The data analysis was performed using Real Statistics Resource Pack software (Version 7.3.2), Copyright (2013–2021), Charles Zaiontz (www.real-statistics.com (accessed on 27 August 2023)).

## 5. Conclusions

The present study investigated the impact of acute and chronic arsenite exposure on global DNA methylation, gene expression profiles, and cell cycle regulation in human liver cells. The results demonstrate pronounced differences between the short- and long-term treatment of HepG2 cells, providing insight on time-dependent changes in the cellular response. Thus, clear time- and concentration-dependent effects were observed at the transcriptional level for genes involved in metal homeostasis, the oxidative stress response, cell cycle regulation, and DNA repair. Regarding genes related to epigenetic regulation, both long-term exposure periods of 10 and 20 days resulted in significant inductions of *DNMT1* and *DNMT3B*, with time-dependent amplifying effects. Interestingly, under the same conditions, significant concentration-dependent hypomethylation was observed during both long-term exposures, with a pronounced effect already occurring at a concentration of 0.5 µM NaAsO_2_ after 20 days of exposure. In contrast, no impact on global methylation levels was observed during the short-term exposure of 24 h. Global hypomethylation can contribute to malignant transformation by promoting chromosomal defects that lead to genetic instability and spontaneous mutations. Since the even up-regulation of two DNMT-coding genes was observed, our data rather point towards the inhibition of DNMTs at the protein level or a potential co-factor deficiency caused by arsenic metabolism. For example, the zinc-binding structures of DNA methyltransferases may represent potentially sensitive targets; this aspect needs to be further investigated.

## Data Availability

The data presented in this study are available on request from the corresponding author (A.H.) for researchers of academic institutes who meet the criteria for access to the confidential data.

## Supplementary Materials

The following supporting information can be downloaded at: https://www.mdpi.com/article/10.3390/ijms242015238/s1.

## Abbreviations

As3MT	arsenite methyltransferase
dCMP	desoxycytidin-5′-monophosphat
DMA III	dimethylarsinic acid
DNMT1	DNA methyltransferase 1
DNMT3A	DNA methyltransferase 3A
DNMT3B	DNA methyltransferase 3B
GSH	glutathione
HPLC	high-performance liquid chromatography
IARC	International Agency for Research on Cancer
MMA III	monomethylarsonic acid
MT	metallothionein
SAM	S-adenosylmethionine
TXN	thioredoxin
