# Supplementary material for: Effect of Long-Term Low-Dose Arsenic Exposure on DNA Methylation and Gene Expression in Human Liver Cells
Source: Int J Mol Sci. 2023 Oct 16;24(20):15238. doi: 10.3390/ijms242015238 (PMC10607230; doi:10.3390/ijms242015238)
Supplement: Supplementary file 1 [file ijms-24-15238-s001.zip › ijms-2576377-supplementary.pdf]

Supplementary information to:

Effect of long-term low-dose arsenic exposure on DNA methylation and gene expression in human liver cells

Sandra Stößer, Tatjana Lumpp, Franziska Fischer, Sarah Gunesch, Paul Schumacher and Andrea Hartwig*

Department of Food Chemistry and Toxicology, Institute of Applied Biosciences (IAB), Karlsruhe Institute of Technology (KIT), 76131 Karlsruhe, Germany.

* Correspondence: andrea.hartwig@kit.edu

Supplementary Table S1: Genes and the respective encoded proteins analysed in the present study. Genes marked with * are reference genes.

Gene	Encoded protein
*ACTB**	β-actin (ACTB)
APAF1	apoptotic protease activating factor (APAF1)
ATM	ataxia telangiectasia mutated (ATM)
ATR	ataxia telangiectasia and Rad3-related protein (ATR)
*B2M**	beta-2-Microglobulin (B2M)
BAX	bcl2-associated x protein (BAX)
BRCA1	breast cancer 1, early onset (BRCA1)
BRCA2	breast cancer 2, early onset (BRCA2)
BTRC	transducin repeat containing E3 ubiquitin protein ligase, beta (β-TrCP)
CAT	catalase (CAT)
CCL22	C-C motif chemokine ligand 22 (CCl22)
CCND1	cyclin D1 (CCND1)
CDKN1A	cyclin-dependent kinase inhibitor 1A (p21)
CDKN1B	cyclin-dependent kinase inhibitor 1B (p27)
CDKN2A	cyclin-dependent kinase inhibitor 2A (p16)
CDKN2B	cyclin-dependent kinase inhibitor 2B (p15)
CDKN2D	cyclin-dependent kinase inhibitor 2D (p19)
COX2	cytochrome C oxidase assembly factor (COX2)
DDB2	damage-specific DNA binding protein 2 (DDB2)
DDIT3	DNA damage inducible transcript 3 (DDIT3)
DNMT1	DNA methyltransferase 1 (DNMT1)
DNMT3A	DNA methyltransferase 3A (DNMT3A)
DNMT3B	DNA methyltransferase 3B (DNMT3B)
E2F1	E2F transcription factor 1 (E2F1)
EGFR	epidermal growth factor receptor (EGFR)

EHMT2/G9a	euchromatic histone lysine methyltransferase 2(EHMT2)
EP300	E1A binding protein P300 (EP300)
ERCC2	excision repair cross-complementation group 2 (XPD)
ERCC4	excision repair cross-complementation group 4 (XPF)
ERCC5	excision repair cross-complementation group 5 (XPG)
FOXO1	forkhead box O1 (FOXO1)
FOXO3	forkhead box O3 (FOXO3)
FTH1	ferritin heavy chain 1 (FTH1)
G6PD	glucose-6-phosphate dehydrogenase (G6PD)
GADD45A	growth arrest and DNA-damage-inducible, alpha (GADD45A)
*GAPDH**	glyceraldehyde-3-phosphate dehydrogenase (GAPDH)
GCLC	glutamate-cysteine ligase, catalytic subunit (GCL)
GPX1	glutathione peroxidase 1 (GPX1)
GPX2	glutathione peroxidase 2 (GPX2)
GSR	glutathione reductase (GSR)
*GUSB**	glucuronidase, beta (GUSB)
HDAC1	histone deacetylase 1 (HDAC1)
HDAC10	histone deacetylase 10 (HDAC10)
HDAC2	histone deacetylase 2 (HDAC2)
HDAC3	histone deacetylase 3 (HDAC3)
HMOX1	heme oxygenase (decycling) 1 (hMO-1)
*HPRT1**	hypoxanthine phosphoribosyltransferase 1 (HPRT1)
HSPA1A	heat shock 70kDa protein 1A (HSP70)
IL1a	interleukin 1 alpha (IL1a)
IL1b	interleukin 1 beta (IL1b)
IL6	interleukin 6 (IL6)
IL8	interleukin 8 (IL8)
JUN	jun proto-oncogene (c-JUN)
KDM3A	lysine demethylase 3A (KDM3A)
KEAP1	kelch-like ECH-associated protein 1 (KEAP1)
LIG1	ligase I, DNA, ATP-dependent (LIG1)
LIG3	ligase III, DNA, ATP-dependent (LIG3)
MAP3K5	mitogen-activated protein kinase kinase kinase 5 (MAP3K5/ASK1)
MBD4	methyl-CpG binding domain 4 (MBD4)
MDM2	MDM2 Proto-Oncogene (MDM2)
MeCP2	methyl-CpG binding protein 2 (MeCP2)
MGMT	O-6-methylguanine-DNA methyltransferase (MGMT)
MLH1	mutL homolog 1 (MLH1)
MSH2	mutS homolog 2 (MSH2)
MT1X	metallothionein 1X (MT1X)
MT2A	metallothionein 2A (MT2A)
MYC	v-myc avian myelocytomatosis viral oncogene homolog (c-MYC)

NFKB1	nuclear factor of kappa light polypeptide gene enhancer in B-cells 1 (p50/p105)
NFKB2	nuclear factor of kappa light polypeptide gene enhancer in B-cells 2 (p49/p100)
NFKBIA	nuclear factor of kappa light polypeptide gene enhancer in B-cells inhibitor,alpha (IKBA)
OGG1	8-oxoguanine DNA glycosylase (hOGG1)
PARP1	poly (ADP-ribose) polymerase 1 (PARP1)
PLK3	polo-like kinase 3 (PLK3)
PMAIP1	phorbol-12-myristate-13-acetate-induced protein 1 (NOXA)
PRDX1	peroxiredoxin 1 (PRX1)
RAD50	RAD50 homolog (S. cerevisiae) (RAD50)
RAD51	RAD51 recombinase (RAD51)
RRM2B	ribonucleotide reductase M2B (TP53 inducible) (p53R2)
SETD2	SET Domain Containing 2 (SETD2)
SIRT2	sirtuin 2 (SIRT2)
SLC30A1	solute carrier family 30 (zinc transporter), member 1 (ZnT1)
SOD1	superoxide dismutase 1, soluble (SOD1)
SOD2	superoxide dismutase 2, mitochondrial (SOD2/MnSOD)
TET1	tet methylcytosine Dioxygenase 1 (TET1)
TET2	tet methylcytosine Dioxygenase 2 (TET2)
TET3	tet methylcytosine Dioxygenase 3 (TET3)
TGFb	transforming growth factor beta (TGFb)
TNFa	tumor necrosis factor a (TNFa)
TNFRSF10B	tumor necrosis factor receptor superfamily, member 10b (DR5)
TXN	thioredoxin (TXN)
TXNRD1	thioredoxin reductase 1 (TXNRD)
VEGFA	vascular endothelial growth factor A (VEGFA)
XPA	xeroderma pigmentosum, complementation group A (XPA)
XPC	xeroderma pigmentosum, complementation group C (XPC)
XRCC5	X-ray repair complementing defective repair in Chinese hamster cells 5 (XRCC5)

Supplementary Table S2: Detailed information on the primers newly established in addition to the existing gene set [16].

Gene	RefSeq		Sequence 5` --> 3`	Product size [bp]	Annealing Tm (°C)
CDKN2A	NM_000077	fwd:	CCCACTACCGTAAATGTCCATT	242	66.5
		rev:	GACTCAAGAGAAGCCAGTAACC		
CDKN2D	NM_001800	fwd:	TTGTGGCTTATAGGTGTTGGTT	126	66.4
		rev:	ACCGTTTAGGTGGCTGTG		
DNMT1	NM_001130823	fwd:	GGCAAACCACCATCACATCTCATT	162	67.9
		rev:	GCGGTCTAGCAACTCGTTCTCT		
DNMT3A	NM_022552	fwd:	CGGCAAATTCTCAGTGGTGTGT	122	67.8
		rev:	GCAGGACCTCGTAGATGGCTT		
DNMT3B	NM_006892	fwd:	CTCCTCCATATCTCCCTCTTCCCTA	181	67.9
		rev:	ACTGCTGTCACTGCCTCTGA		
EHMT2	NM_006709	fwd:	TCCGCATGAGTGATGATGTCCA	223	68
		rev:	CGCTCATCCACAGAGTAGGAATCA		
EP300	NM_001429	fwd:	GTGCTGGCAACTTACTGACTGAG	165	67.6
		rev:	CACTGGCTCCAATCTGCTGTC		
FOXO1	NM_002015	fwd:	ACCCAGCCCAAACTACCA	161	67.6
		rev:	AGTCAGAAGTCAGCAACTCCTT		
FOXO3	NM_001455	fwd:	GTGTTCGGACCTTCATCTCTG	149	66.8
		rev:	TCGCTGTGGCTAAGTGAGT		
HDAC1	NM_004964	fwd:	GGGTCAAGGAGGAGGTCAAGTT	84	67.5
		rev:	GGACTCAGCAGGAAGCCAGA		
HDAC10	NM_032019	fwd:	CTCTTAGATGGGATGCTGGATGG	200	66.4
		rev:	CTGATGTTCAGCCACAGACTCC		
HDAC2	NM_001527	fwd:	GCCAGTTAGTATCAGTGAAGATTAACATCC	173	67.5
		rev:	TAGGCAAGTCTGGTCAATACAAGTTCT		
HDAC3	NM_001355039	fwd:	CCACTCCGAGGACTACATTGACT	75	67.6
		rev:	TTAAGACTCTTGGTGAAGCCTTGC		
KDM3A	NM_001146688	fwd:	TGTGAGGAGATTCCAGCACTGAA	106	67.8
		rev:	CAGCTCCAATTCTTGCAGAATTACCA		
MBD4	NM_003925	fwd:	GCCGAATGACCTCCGCAAAG	126	68.2
		rev:	GCAGAAGCGATGGGTTCTTGTAG		
MeCP2	NM_004992	fwd:	CCGCTCTGCTGGGAAGTATGA	192	68.2
		rev:	GATTTGGGCTTCTTAGGTGGTTTCTG		
SETD2	NM_014159	fwd:	CCCGACCCCTGAAGAAGAAGA	176	67.6
		rev:	GGCTGAAGCTGAATGACACCTT		
TET1	NM_030625	fwd:	AACCTTAGGGAGTAACACTGAGACC	114	67.5
		rev:	GCGGATGGCATCAGCGAAT		
TET2	NM_001127208	fwd:	CCTGTGATGCTGATGATGCTGATAA	195	67.2
		rev:	AATTGCTGCTGGAACCTGAACA		
TET3	NM_001366022	fwd:	TCAAGGTGGAGCCGCAGAA	130	68.1
		rev:	TGGTAGGAGTACACGCTGTTCAT		

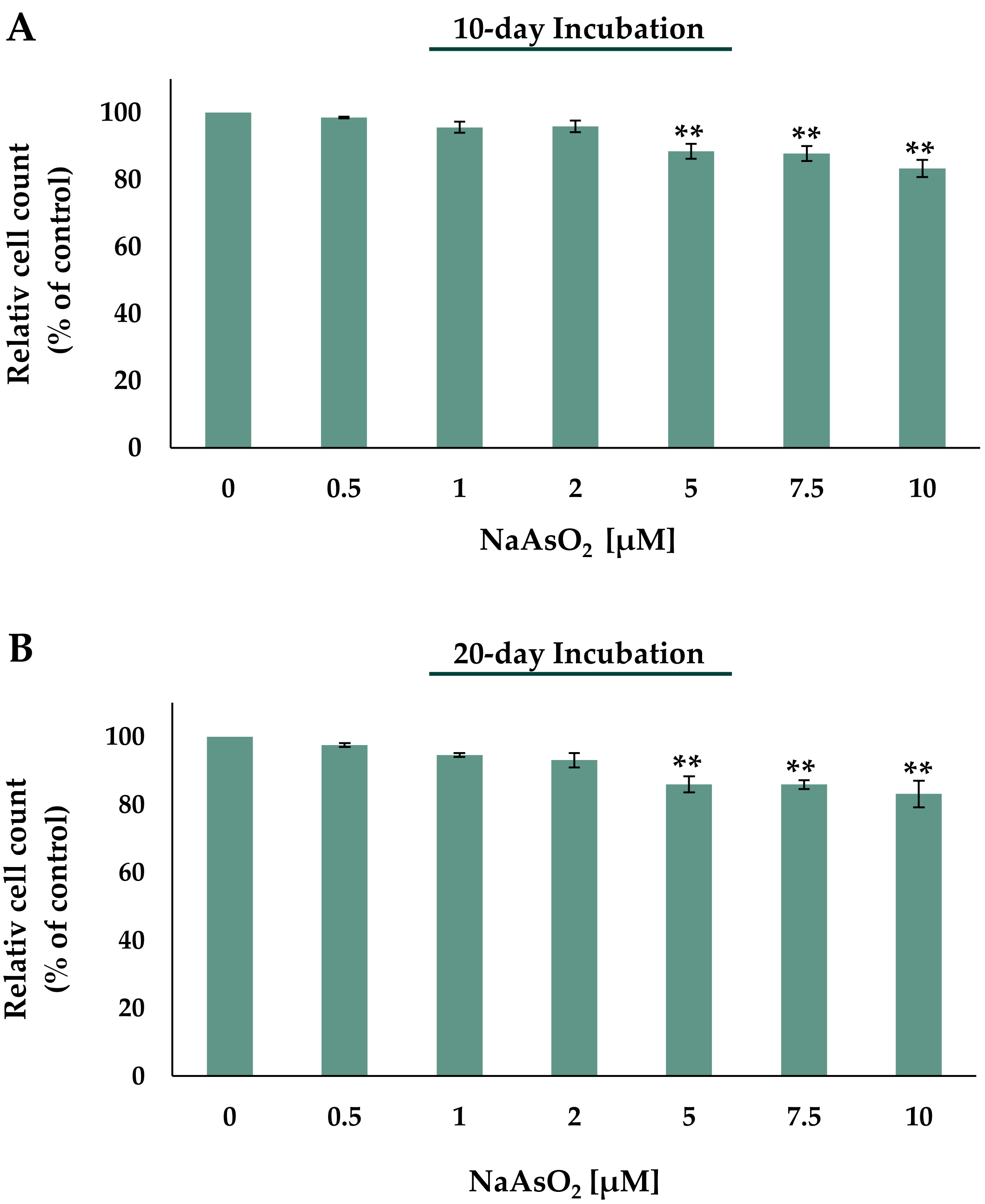

Figure S1: Relative cell counts of HepG2 cells after 10-day (A) as well as 20-day (B) exposure to $NaAsO_2$. Mean values ± standard deviations (SD) from three independent experiments are shown. Statistical analysis was performed to determine the differences between the exposed cells and the negative control using one-way ANOVA followed by Dunnett's post hoc test: ** ($p \leq 0.01$).

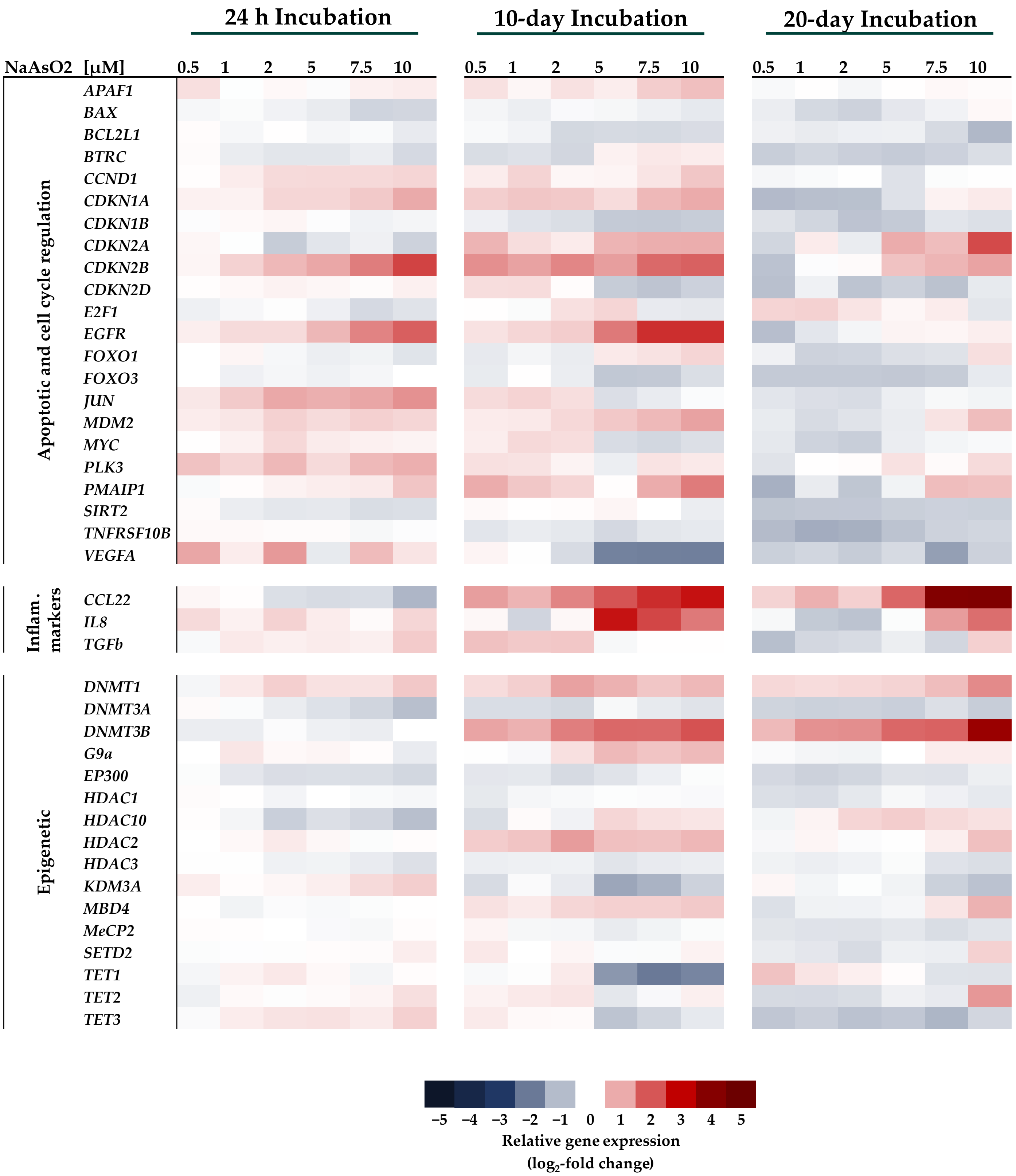

Figure S2: Gene expression profile of HepG2 cells after 24-hour, 10-day, and 20-day treatment with $NaAsO_2$. The clusters of genes involved in cell cycle, apoptotic and senescence, inflammation and epigenetic regulation are presented. The log_2-fold changes are provided for three independent experiments, normalized to the untreated control, where the control is set to 0.

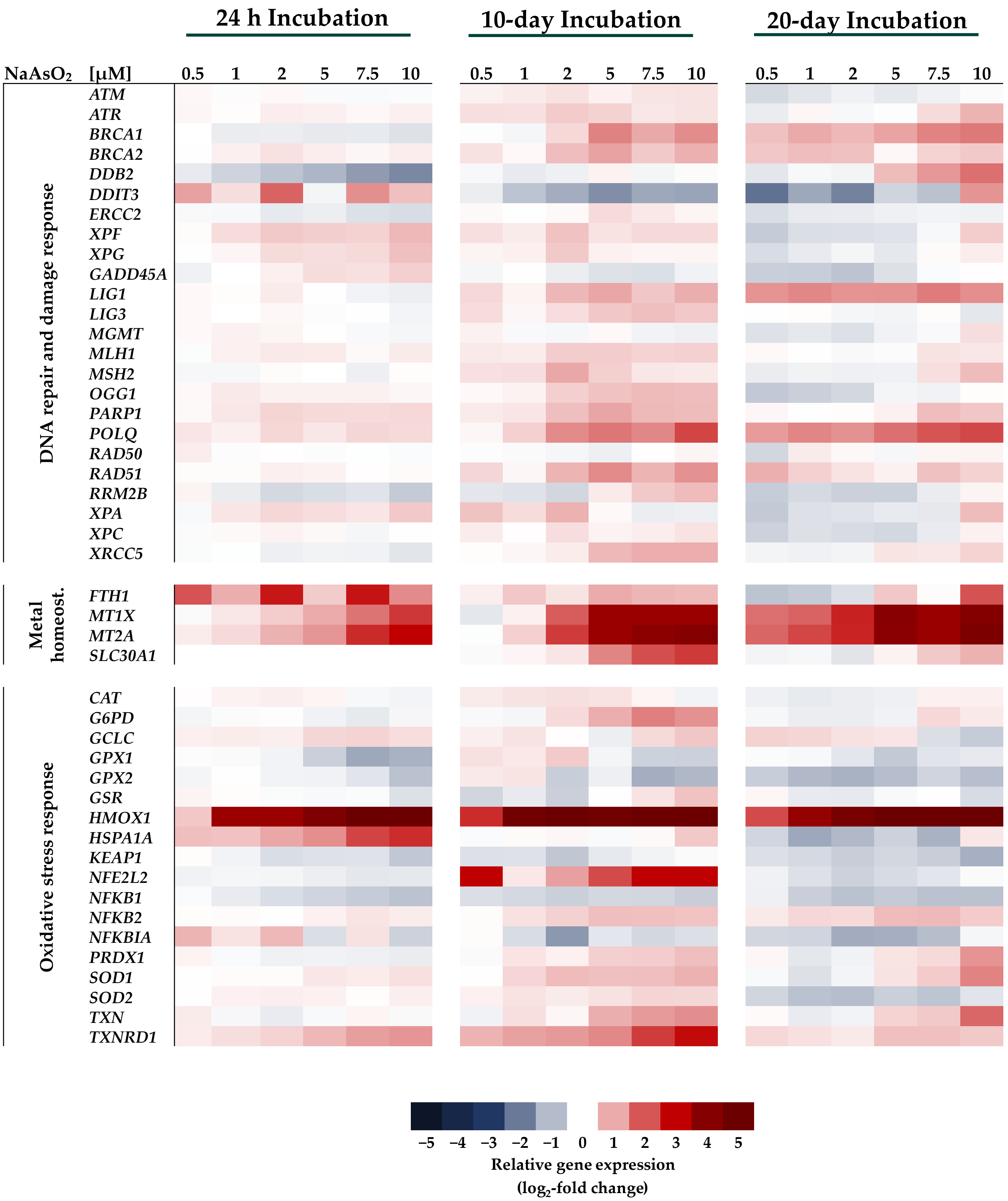

Figure S3: Gene expression profile of HepG2 cells after 24-hour, 10-day, and 20-day treatment with $NaAsO_2$. The clusters of genes involved in metal homeostasis, response to oxidative stress, and DNA damage response are presented. The log_2-fold changes are provided for three independent experiments, normalized to the untreated control, where the control is set to 0.
